# Molecular Weight Distribution of Polymeric Proteins in Wheat Grains: The Rheologically Active Polymers

**DOI:** 10.3390/foods9111675

**Published:** 2020-11-16

**Authors:** Thierry Aussenac, Larbi Rhazi, Gérard Branlard

**Affiliations:** 1 Institut Polytechnique UniLaSalle, Université d’Artois, URL 7519, 19 rue Pierre Waguet, BP 30313, 60026 Beauvais, France; larbi.rhazi@unilasalle.fr; 2 INRAE, UCA UMR1095 GDEC, 5 Chemin de Beaulieu, 63100 Clermont-Ferrand, France; gerard.branlard@gmail.com

**Keywords:** wheat, bread making quality, MWD of polymeric proteins, environmental effects on HMW polymers

## Abstract

We characterized the molecular weight distribution of polymeric proteins (PP) of bread wheat grains using asymmetric flow field flow fractionation (A4F). The experiment, involving six environmental conditions and 130 cultivars, offered the opportunity to approach the phenotypic values of the polymer characteristics and their contribution of the rheological properties of flours and/or doughs. The contents of high-molecular-weight polymers (M_W_ > 2 × 10^6^ g·mol^−1^) that can be considered as “rheologically active polymers” (RAPP) for their major contribution to dough baking strength and mixing tolerance were mainly controlled by environmental factors. Under the influence of the growing conditions, at the cellular level, the redox status of non-protein free thiol, such as glutathione, is modified and leads to the formation of polymeric protein-bound glutathione conjugates (PPSSG). The accumulation of these conjugates reduces the formation of the RAPP by limiting the intermolecular interactions between PP in the grain during desiccation. This phenomenon is, therefore, potentially responsible for decreases in the technological properties of the wheat genotypes concerned. These first results invite us to continue our investigations to fully confirm this phenomenon, with emphasis on the behavior of wheat genotypes under various growing conditions.

## 1. Introduction

During the last 60 years, in the field of cereal chemistry, the scientific community has been working to determine, in an ever more precise way, the nature of the constituents responsible for the acquisition of technological properties (i.e., breadmaking properties for doughs of bread wheat (*Triticum aestivum*) and/or pasta properties of durum wheat (*Triticum durum*)). Emphasis has been placed on those whose (quantitative and/or qualitative) variations account for observed and measured changes in processing ability.

Nowadays, a broad consensus is emerging to affirm that the value of use of a flour largely depends on the quality of its prolamins assembly which is, in turn, influenced by protein polymorphism [1,2,3] and the general conditions of grain development and maturation [4,5,6,7,8,9,10,11]. The molecular weight distribution (MWD) of storage proteins includes both the relative proportions of monomeric proteins (mainly represented by gliadins) and polymeric proteins (glutenins) [12], but also the degree of association of polymeric proteins [13,14].

Since the 1990–2000s, the level of association of glutenins in endosperm or in flour has been estimated from the level of solubility of these polymers, in particular using dissociative solvents such as 2% (*w*/*v*) SDS. This practice facilitates the qualification of polymers as “easily extractable” and as “difficult to extract—UPP” [13], “insoluble” [15], or even “GMP” (glutenin macropolymer) [16]. Numerous authors have been able to relate this notion of polymeric distribution within pastes and gluten with their respective rheological behaviors [2,13,14,17]. These studies all agree in presenting “insoluble” gluten polymers or even “GMP” as the main actors responsible for the observed rheological modifications [18]. Finally, and in the absence of reliable and relevant analytical methodologies, a description of the specific role of high-molecular polymers (i.e., polymers whose molecular dimensions are greater than a certain critical value) has been presented by several authors [19,20,21] as a hypothesis and by analogy with the theory of Bersted and Anderson [22].

Considerable advances have been made over the last 30 years at a genetic level to better understand and/or to promote the interaction properties of prolamins (i.e., genetic control of the MWD of polymeric proteins, particularly by manipulating the allelic diversity of High Molecular Weight glutenin subunits (HMW-GS)). However, it now appears that genetic variations of glutenins had less influence than environmental factors on polymer characteristics [23]. Irrespective of the environmental factors studied (i.e., nitrogen availability, sulfur fertilization, temperature and water regime), in most of these works, the effects observed are most often attributed to modifications in the synthesis activities of the various prolamins [i.e., gliadins vs. glutenins and/or HMW-GS vs. Low Molecular Weight glutenin subunits (LMW-GS)] resulting from modulation of the expression of storage protein genes [24]. Today, it seems that other phenomena could also be reasonably involved in explaining the environmental control of the accumulation of high-molecular polymers during the grain desiccation phase. In our hypothesis, these phenomena could be based in particular on significant variations in the cellular redox status in response to environmental stimuli (i.e., various environmental stresses); these variations can lead to a significant decrease in the degree of association of the polymeric proteins by limiting the protein–protein interactions (i.e., formation of intermolecular disulfide bonds from free sulhydryl (SH) residues) during the desiccation of the grains [9,10].

Considering the current and projected environmental impacts (i.e., climate changes with increasing heat and/or water stresses in particular), it is essential to better understand these phenomena to implement new breeding strategies for a sustainable quality. Consequently, based on a large panel of French wheat genotypes grown in contrasting environments, our study aims to (i) better characterize the role of the polymeric protein fraction in the definition of the rheological properties of wheat flours/doughs, (ii) to prioritize the main factors influencing the formation of the polymers of interest as previously defined; and finally (iii) to highlight the potential effects of some cellular free thiols, in particular glutathione, on the molecular weight distribution of polymeric proteins during grain desiccation.

## 2. Materials and Methods

### 2.1. Multilocal Trials and Plant Material

In the frame of collaborative studies between INRA and the majority of European private breeding companies operating in France, multilocal trials have been carried out during 2004–2006 (Program “Indices of Quality”). UniLaSalle was associated with these studies led by INRA and financed by the FSOV (i.e., Fond de Soutien à l’Obtention Végétale). Many biochemical, genetic, and technological parameters were measured on the 780 collected samples (130 cultivars in 6 locations in total; Supplementary Table S1). The main results and the presentations given can be consulted (https://www.fsov.org).

Experimental trials were located in the major areas of wheat production in France. Each trial was conducted in a field experiment including randomized plots (10 m^2^) with two replicates and using conventional agronomic practices, with mineral fertilization and fungicide protection, to achieve high grain yields without any water irrigation. The 130 cultivars, originating mostly from France but also from some other European countries (UK, The Netherland, Germany), were hexaploid winter bread wheat cultivars (registered varieties or homozygous stable cultivars) proposed by INRA and associated breeding companies. They were not pedigree related (sister lines). The allelic diversity of HMW-GS and LMW-GS encoded at the three *Glu-1* and *Glu-3* loci was identified for the 130 cultivars studied. Proteins were extracted from 5 to 10 individual seeds for each cultivar and submitted to SDS-polyacrylamide gel electrophoresis as described by Branlard et al. [25]. The alleles identified according to the previously used nomenclature are given in Supplementary Table S2.

### 2.2. Sample Preparation, Total Protein Content Determination, and Technological Tests

For each cultivar, the grains harvested from the two plots after cleaning were blended (50/50 *w*/*w*) and the resulting sample (approx. 1000 g) was maintained for at least 3 weeks at 15 °C and 65% relative hygrometry for moisture homogenization. Fifty grams of grains were milled using a Cyclotec^TM^ 1093 (FOSS, Hillerød, Denmark) with a sieve size of 0.75 mm. The wholemeal was used for total protein concentration determination (TPC). The total protein content (TPC) of each sample was measured on 120 mg of oven-dried (80 °C for 48 h) wheat flour by elementary nitrogen analysis (DUMAS method—AOAC 7024) on a Leco apparatus (model FP528). Three replicates were carried out and combined for analysis, using a conversion factor of N × 5.7. For each wheat sample, approximately 20 g of wholemeal was kept in a closed vial at −20 °C for polymer extractions and analysis.

A 10 g Mixograph (National manufacturer, Lincoln, NE, USA), with the Mixmart^®^ software according to the AACC method 58-40 A [26], was used to register the consistency during dough mixing. Flour preparation, dough hydration, and the parameters used are described in Martinant et al. [27]. The alveograph test (method AACC 54-30A) was carried out on about 70% extraction rate flour obtained using the CD2 Chopin mill. The rheological dough parameters strength (W), tenacity (P), and extensibility (L) were computed using the Chopin Alveolink apparatus (Tripette & Renaud, Villeneuve La Garenne, France). Some aspects of the rheological quality of these multilocal trials have previously been reported by Oury et al. [28].

### 2.3. Characterization of the Molecular Weight Distribution (MWD) of Grain Storage Proteins by Asymmetric Flow Field-Flow Fractionation (A4F)

Samples were prepared according to the protocol for protein extraction described in detail by Lemelin et al. [11]. Briefly, wholemeal samples (30 mg) were dispersed and incubated at 60 °C for 15 min with 1.0 mL of 0.05 M sodium phosphate buffer (pH 6.9) containing 2% (*w*/*v*) SDS. The samples were then sonicated for 20 s at a power setting of 30%. The supernatant (centrifugation at 12,500 × g at 20 °C for 15 min) was filtered through a syringe filter of regenerated cellulose of 0.45 µm (Gelman Sciences, Pall France, Saint-Germain-en-Laye, France) before injection (30 µL) into the A4F/MALLS system. Protein concentration was determined for each extract by combustion nitrogen analysis, as described above. The A4F was accomplished with an Eclipse3 F System (Wyatt Technology, Santa Barbara, CA, USA) serially connected to a UV detector (Agilent 1200, Agilent Technologies, Waldbronn, Germany), a MALLS detector (Dawn multiangle Heleos TM, Wyatt Technology Corporation, Toulouse, France), and an interferometric refractometer (Optilab rEX, Wyatt Technology Corporation, Toulouse, France). Absorbance was measured at 214 nm. The channel had a trapezoidal geometry and a length of 286 mm. The thickness of the spacer used in this experiment was 350 mm. The ultrafiltration membrane forming the accumulation wall was made of regenerated cellulose with a cut-off of 10 kDa (LC-10 Nadir reg. Cell, Wyatt Technology Europe, Dernbach, Germany). An Agilent 1200 Series Isocratic HPLC Pump (Agilent Technologies, Waldbronn, Germany) with an inline vacuum degasser delivered the carrier flow to the channel. A 0.45 µm in-line filter (Gelman Sciences, Pall France, Saint-Germain-en-Laye, France) was installed between the main pump and the Eclipse system. Sodium phosphate buffer (0.05 M, pH 6.9) containing 0.1% (*w*/*v*) SDS was used as the mobile phase and filtered through a 0.1-µm membrane (Gelman Sciences, Pall France, Saint-Germain-en-Laye, France). For the fractionations using a gradient in the cross-flow, the focus time was 0.5 min at a flow rate of 2 mL min^−1^, followed by a focus/injection time of 1.0 min at 0.2 mL min^−1^ and a relaxation/focusing period of 0.5 min. Elution then followed at an outflow rate (Fout) of 1.0 mL min^−1^ and with a cross-flow rate (Fc) decreasing exponentially from 3.0 to 0.0 mL min^−1^ for 14 min. Finally, elution at a cross-flow rate of 0.0 mL min^−1^ was maintained for 9 min. A bovine serum albumin (BSA) standard was used to normalize the diodes of the MALLS detector. All necessary constants for molecular weight distribution calculation (weight-average molar mass (Mw), number-average molar mass (Mn), polydispersity (Mw/Mn), number-average mean square radius (Rn), and weight-average mean square radius (Rw)) were determined as previously described [11].

A correlation study was conducted to determine the impact of the different molecular masses of polymers on the performance of technological variables. The values of the technological variables [Midline Peak Width MPW), Midline Left of Peak Width (MLW), Midline Time Integral (MT × I), Midline Time Width (MT × W), Dough baking strength (W), Dough viscoelastic balance (P/L)] obtained on all 780 samples are correlated with the MWD values of the polymers acquired every 0.003 min during the A4F measurement of the proteins extracted on the wholemeal of the same samples.

### 2.4. Extraction and Derivatization of the Polymeric Protein-Bound Glutathione (PPSSG)

Quantification of the PPSSG, corresponding to glutenin-bound glutathione, requires extraction and purification of polymeric proteins. The procedure used here was performed according to Rhazi et al. [10] with some modifications. Wholemeal samples (0.125 g) were stirred for 1 h at room temperature (25 °C) with 2.25 mL of 0.08 M Tris-HCl buffer (pH 7.5) containing 50% (*v*/*v*) 1-propanol. Extraction was followed by centrifugation (15,900× *g* for 30 min at 15 °C). Soluble polymeric proteins in 50% 1-propanol were precipitated by the addition of 1.5 mL of 1-propanol to bring the 1-propanol concentration of the supernatant to 70% (*v*/*v*). The dispersion was agitated and left to stand at room temperature for 1 h after centrifugation at 26,000× *g* for 15 min at 15 °C; the supernatant, which mainly contained monomeric proteins, was removed. The pellet, containing mainly PP and PPSSG, was mixed with 1 mL of the Tris-DTT solution (0.2 M Tris, 0.025 M DTT, pH 9.0) and then allowed to stand at 40 °C for 1 h. Subsequently, the samples were derivatized as described above.

The derivatization procedure included the reaction of iodoacetic acid (IAA) with thiols to form S-carboxymethyl derivatives, followed by chromophore derivatization (DNP) of primary amines with Sanger’s reagent, 2,4-dinitro-1-fluorobenzene (FDNB). To 1 mL of acid extracts obtained from extraction of free glutathione and protein-bound glutathione, 100 μL of ice-cold phenanthroline solution (50 mM, prepared in absolute ethanol) was added. The mixture was bubbled thoroughly with nitrogen, homogenized briefly, and kept on ice. Then, 100 μL of 0.1M IAA in a 0.2-mM m-cresol purple solution was added to the samples containing phenanthroline. The tubes were bubbled with nitrogen and vortexed. The acidic solution (pink in color) was brought to pH 8–9 (purple colour) by the addition of 105 mg of NaHCO_3_, followed by bubbling with nitrogen and vortexing after closing the lids. Samples were incubated in the dark at room temperature for 1 h, with intermittent vortexing. After incubation, 3% (*v*/*v*) FDNB solution freshly prepared in absolute ethanol (0.75 mL) was added, and the reaction mixture was capped, briefly mixed, and stored at room temperature overnight. Finally, after centrifugation at 15,900× *g* at 20 °C for 15 min, samples were filtered through 0.22-μm hydrophilic polypropylene membranes (AcroPrep GHP, Gelman Sciences, Pall France, Saint-Germain-en-Laye, France) and stored in the dark at 4 °C until high performance liquid chromatography (HPLC) analysis.

### 2.5. HPLC Quantification of the Polymeric Protein-Bound Glutathione (PPSSG)

The HPLC analysis was carried out using a Thermo Scientific Surveyor Plus HPLC System (Thermo Separation Products S.A., Les Ulis, France); the data were recorded on a ChromQuest 5.0 data system (Thermo Separation Products S.A., Les Ulis, France). Dinitrophenylated derivatives were separated on a Spherisorb amino bonded analytical column (250 × 4.6 mm i.d., 5 μm particle size) (CIL, Cluzeau, Sainte-Foy-la-Grande, France). Solvents for HPLC were as follows: A, 4/1 (*v*/*v*) methanol/water; B was prepared by dissolving sodium acetate trihydrate (272 g) in water (122 mL) and glacial acetic acid (378 mL) and mixing the resultant solution (200 mL) with solution A (800 mL). After injection of the sample (0.1 mL), the column was eluted with 85% solvent A/15% solvent B for 10 min. A linear gradient from 15 to 100% solvent B was then applied over a 15 min period. After elution for 3 min at 100% solvent B, the proportion of this solvent was reduced linearly to 15% over 2 min. During the separation, the solvent flow rate was maintained at 0.7 mL min^−1^. The column effluent was monitored at 365 nm.

### 2.6. Statistical Analysis

All statistical analyses (i.e., descriptive statistics, simple regression, Pearson’s correlation, and ANOVA with the general linear model (GLM)) were performed using the Statgraphics^®^ software (FRANCESTAT, Neuilly sur Seine, France). Statistical significance was accepted at *p* < 0.01 (*), *p* < 0.001 (**), and *p* < 0.0001 (***).

## 3. Results and Discussion

### 3.1. MWD of Polymeric Proteins Is a Key Factor for Bread-Making Quality

The genetic diversity of the subunits of glutenins was first analyzed in the 130 cultivars of the six locations. In total, 31 alleles (14 encoding the HMW-GS at *Glu-1* loci and 17 encoding the LMW-GS at *Glu-3* loci) were identified (Table S2). This high level of genetic diversity of glutenin subunits resulted from the origin of the European bread wheat cultivars involved in our multilocal trials. The cultivars of the six locations were homozygous at each loci encoding glutenin subunits and the genetic diversity remained unchanged over 2 consecutive years. Polymers resulting from the polymerization of these glutenin subunits were precisely characterized in each location for each cultivar.

In view of this objective, we implemented the analytical methodology (A4F/MALLS) that, as we have demonstrated in a previous work [25], allows us to go beyond conventional approaches used, such as Size Exclusion HPLC (SE-HPLC) [13,15] or even differential solubility in buffered solutions containing SDS [29,30].

**Figure 1:**
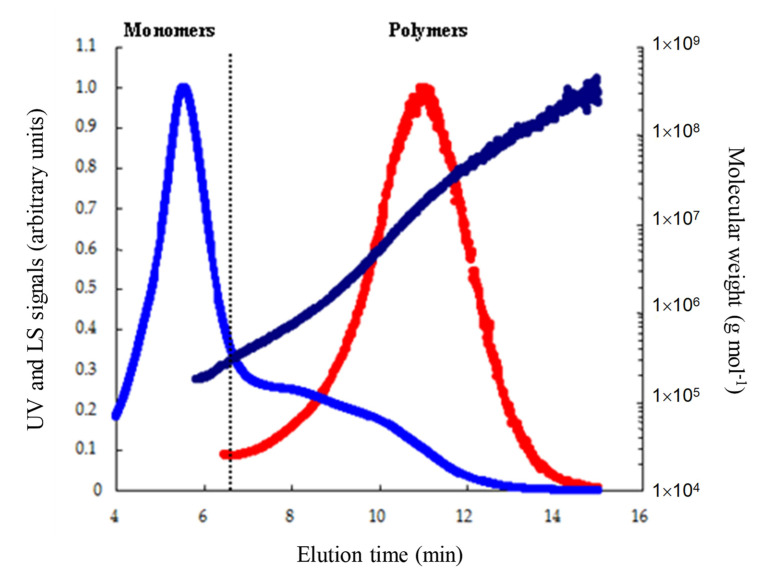
Asymmetrical flow field-flow fractionation (A4F) profiles of total solubilized storage proteins of a French common wheat cultivar (*Soissons*). UV detection at 214 nm (blue line), light scattering signal at 90° (red line), and calculated molecular mass as a function of elution time (dark blue line). The dotted line materializes the theoretical limit between fractions of monomeric proteins vs. polymeric proteins.

Indeed, even if these methods provide indications on the molecular distribution of prolamins, they remain rather coarse and even present, in some cases, real limits of interpretation. Conversely, the analytical protocol used here allows us to separate, characterize, and quantify the different protein fractions of wheat grain without a limitation of size and/or conformation and without needing a reference to conventional calibration methods (based on retention times), but using the light scattering (LS) properties of the particles. As the results clearly illustrate in Figure 1 (UV signal vs. LS signal), the methodology used makes it possible to obtain a highly significant discrimination (linear variation of molecular masses as a function of elution time) of the protein fractions extracted; the molecular masses obtained were generally between 5 × 10^4^ g mol^−1^ (various monomers) and more than 5 × 10^8^ g mol^−1^ (polymeric proteins).

**Figure 2:**
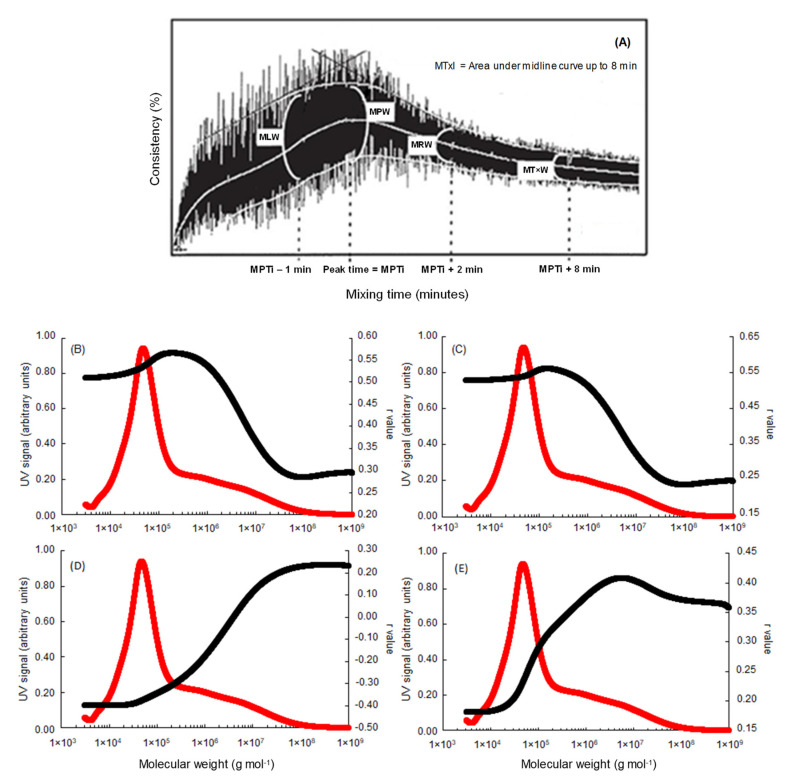
Representation of the different mixogram parameters used (**A**) and plot of simple linear correlation coefficients (*n* = 780) between A4F data (weight-average molecular mass, Mw) and mixogram parameters: MPW (**B**), MLW (**C**), MT × I (**D**), and MT × W (**E**). The UV signal (214 nm), which is represented by the red line, corresponds to the mean fractogram of the 130 wheat genotypes studied.

In a second step, we compared the values of the molecular mass (weight-average molecular mass, Mw) with the technological data of these 130 European bread wheats grown in France in six experimental trials. This comparison was first performed with the data recorded during mixing the different wheat flour samples using a standardized mixograph. Among the different mixogram parameters recorded, only the most relevant variables (good representation of dough formation with a minimum of redundant information) were selected for the rheological characterization (MLW, MT × I, MPW, MRW, MT × W) of the different flours (Figure 2A). The MPTi parameter (i.e., Mixing Peak Time), which defines the mixing time necessary to obtain the maximum consistency of the dough, serves also as a reference for determining the MLW (i.e., Mixing Left Width) at a mixing time equal to MPTi—1 min and the MRW (i.e., Mixing Right Width) at a mixing time equal to MPTi + 2 min. All these parameters are conventionally used to define the mixing tolerance of the doughs. The MT × W parameter (i.e., Midline Time Width), which defines the mixing tolerance, is measured at a mixing time equal to MPTi + 8min. Finally, the area under the midline curve (MT × I) corresponds to the energy needed to form the glutinous network.

**Figure 3:**
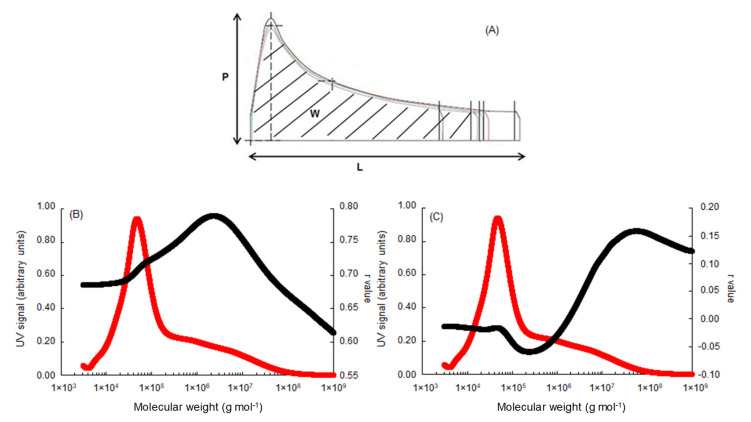
Representation of the different alveogram parameters used (**A**) and plot of simple linear correlation coefficients (*n* = 780) between A4F data (weight-average molecular mass, Mw): W (**B**) and P/L (**C**). The UV signal (214 nm), which is represented by the red line, corresponds to the mean fractogram of the 130 wheat genotypes studied.

The dough behaviors after mixing were evaluated using the standardized Alveograph method measuring the essential rheological characteristics of the dough: (P) represents dough tenacity (i.e., aptitude to resist deformation), (L) is dough extensibility (i.e., maximum volume of air that the bubble is able to contain), and (W) corresponds to dough baking strength (surface under the curve) (Figure 3A). The P/L ratio reflects the viscoelastic balance of the dough (tenacity vs. extensibility).

The areas of the fractograms of all samples (*n* = 130 × 3 × 2 = 780) were integrated at 0.003 min intervals from 15.0 to 4.0 min (for A4F, the order of elution of protein fractions is reversed compared to SE-HPLC) as illustrated in Figure 1. Correlations of MPW, MLW, MT × I, MT × W (Figure 2B–E, respectively), and W, P/L (Figure 3B, C, respectively) against the cumulative areas at each elution time step were then calculated for the complete set of samples. As shown in Figure 2 and Figure 3, even if the different properties of the doughs are characterized by a wide range (Table 1), their variations were significantly influenced by the molecular distribution of the prolamins present.

At the beginning of the mixing procedure, the contents of monomers and the low-molecular-weight polymeric proteins were predominant in their ability to explain MLW and MPW (Figure 2B,C). The behavior of the two mixographic parameters was similar, and maximum r values (≈0.55 ***) were obtained by the same molecular mass (i.e., molecules having a molecular mass ≤2 × 10^5^ g mol^−1^). On the other hand, the high-molecular-weight polymeric protein contents became predominant in their ability to explain mixing tolerance (MRW) (not shown) 2 min after peak time (Figure 2E). At 8 min after the beginning of mixing, the two parameters, mixing tolerance MTxW and the integral MPTxI (Figure 2D,E), were clearly explained by high-molecular-weight polymeric proteins measured by A4F. Maximum *r* values (≈0.40 ***) were obtained for molecules with a molecular mass greater than 6 × 10^6^ g mol^−1^.

The area under the midline curve (MT × I), which is one of the main mixographic parameters and corresponds to the energy needed to form the glutinous network, would be characterized by a molecular mass equilibrium. As presented in Figure 2D, beyond the molecular mass of 2 × 10^6^ g mol^−1^, MT × I is positively correlated with the quantity of polymeric proteins and the r value increases with the increase in molecular mass of these polymers to reach a maximum value of about 2 × 10^8^ g mol^−1^ (*r* ≈ +0.24 ***). The more the mass of the polymers increases, the more the energy supplied to form the glutinous network increases. Conversely, below the molecular weight of 2 × 10^6^ g mol^−1^, the MT × I is negatively correlated with the quantity of monomers and low-molecular-mass polymeric protein, and the value of r increases with the reduction in molecular mass of these proteins to reach a maximum value of about 2 × 10^4^ g mol^−1^ (*r* ≈ −0.40 ***). These negative correlations demonstrate the inability of small polymers to form a glutinous network. The higher variations of the dough consistency (width of the curve 1 min before peak time and at peak time MLW and MPW, respectively) measured during mixing are strongly correlated to polymers of reduced molecular mass (Figure 2B,C).

At the same time, the molecular distribution of prolamins significantly affects the dough baking strength (W) as measured by the Chopin alveograph (Figure 3B). As can be seen from the results presented, the *r* value increases with the decrease in molecular mass of the polymeric proteins from 1 × 10^9^ g mol^−1^; the molecular mass reaches a maximum value of about 2 × 10^6^ g mol^−1^ (*r* ≈ +0.78 ***) and thereafter falls continuously. These results suggest that the content of the polymeric protein characterized by a molecular mass higher than 2 × 10^6^ g mol^−1^ (Or ≈ 41% of total polymeric protein on average in our study) strongly contributes to dough baking strength.

Finally, as perfectly illustrated by the results presented in Figure 3C, the molecular distribution of prolamins widely alters the viscoelastic balance of the dough by shifting the balance between dough tenacity (P) and dough extensibility (L). Monomers and low-molecular-mass polymeric protein contents are predominant in their ability to explain dough extensibility (i.e., negatively correlated with P/L). On the other hand, the high-molecular-weight polymeric protein contents (i.e., molecules with a molecular mass higher than 2 × 10^6^ g mol^−1^) became predominant in their ability to explain dough tenacity (i.e., positively correlated with P/L).

Our results, which are consistent with major observations cited in the literature [2,13,14,17], support the general idea that rheological properties of flours and doughs are largely influenced by the aggregative protein polymers. In the quasi absence of any analytical constraints and/or molecular mass estimations due to the use of the A4F protocol, these results also confirm that a critical molecular mass can be defined as the molecular mass above which the polymeric proteins contribute to dough baking strength and mixing tolerance. Even if this hypothesis has already been mentioned by certain authors [20,21], the estimates made based on their SE-HPLC calibration (i.e., 2.5 × 10^5^ g mol^−1^) are largely below our results. Indeed, according to our observations, this critical molecular mass corresponds to average values of 2 × 10^6^ g mol^−1^ (Figure 2 and Figure 3). This fraction of “rheologically active polymeric proteins” will be called “RAPP” in the remaining part of this paper.

### 3.2. Polymer MWD Responded More to Growing Conditions than Genetic Factors

In the context of our study, the polymeric protein (PP) content is characterized by a large amplitude of variation (i.e., from 2.78 to 5.75 g 100 g, with 4.02 g 100 g as the mean value). As presented in Table 2, polymeric proteins accumulated during wheat grain development are strongly under the control of growing conditions. This also confirm previous analyses showing that allelic diversity of HMW-GS and LMW-GS had less influence than environmental factors, such as temperature during the last month, on polymer characteristics [23]. Indeed, the ratio of variances (i.e., environmental variance/genetic variance) is here largely in favor of an environmental effect (σ^2^_E_/σ^2^_G_ >16.0).

**Figure 4:**
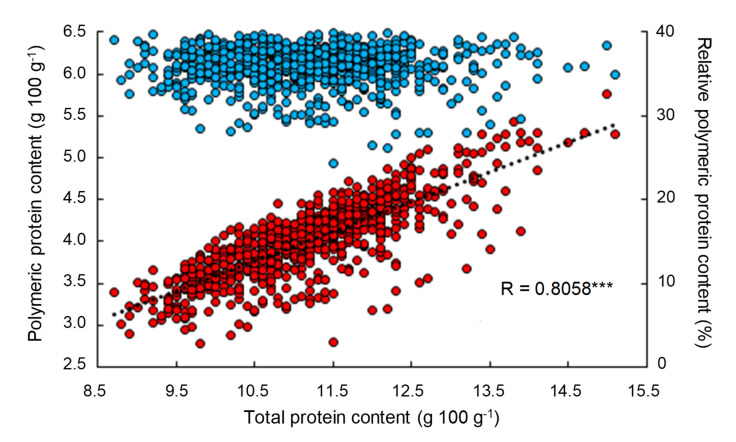
Relationship between protein content and polymeric protein content in wheat grains. Contents expressed in g 100 g of flour (●) or in relative % (●). Statistical significance was accepted at *p* < 0.0001 (***).

As shown by the results presented in Figure 4, among different environmental sources of variation, one of the first causes of modulation of the polymeric protein content of grains is directly linked to the general protein metabolism of the plant from anthesis until the acquisition of physiological maturity (i.e., remobilization phenomenon of the nitrogen reserves from the vegetative parts to the developing grains during the grain-filling period) [31,32,33,34]. The quantity of nitrogen that can be remobilized is, for its part, strongly dependent on the quantity of nitrogen stored during the vegetative period, which is highly dependent on environmental conditions [33,34,35].

Polymeric proteins have molecular characteristics (MW, PI = Mw/Mn and RW) that are mostly under the control of environmental parameters (Table 2), as revealed by the ratio of the calculated variances (σ^2^_E_/σ^2^_G_ = 11.56, 7.83, and 8.01 for MW, PI, and RW, respectively). In addition, there is no significant relationship between the amount of polymers synthesized and accumulated in the grain and their intrinsic molecular characteristics (M_W_, M_W_/Mn, and R_W)_ (Supplementary Figure S1). In fact, all regression curves between the amount of polymers and these characteristics are stable, irrespective of the PP content.

The RAPP content, which represents an important fraction of the PPs (≈41% of total polymeric protein on average in our study), is characterized by a large amplitude of variation for 100 g of flour (i.e., from 0.84 to 3.11 g, with 1.70 g as mean value). Like in the case of PP, the accumulation of HMW polymeric proteins (RAPP) is strongly under the control of environmental factors (Table 2). For these “rheologically active polymeric proteins” (RAPP), the ratio of variances is largely in favor of environmental control (σ^2^_E_/σ^2^_G_ > 20.0). However, and unlike the PP content, the environmental modulation of the RAPP content is not simply the result of the overall synthesis and accumulation of storage proteins and/or PP during the period of grain filling.

### 3.3. Allelic Diversity of Glutenins Had Limited Influence on Polymer Contents and Characteristics

**Figure 5:**
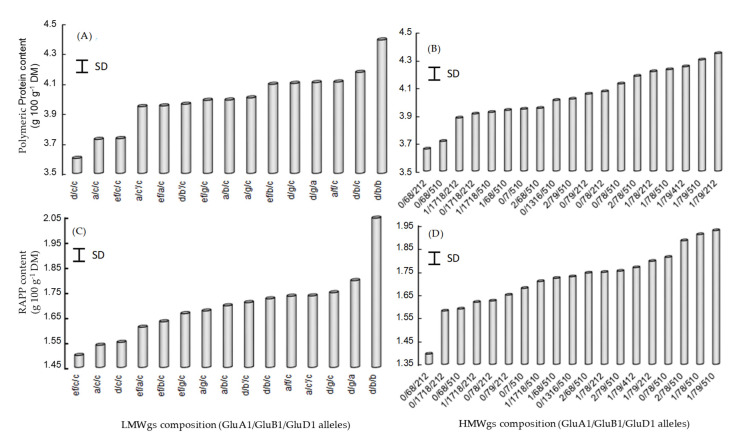
Relationship between (**A**,**C**) LMWgs composition or (**B**,**D**) HMWgs composition of polymeric protein (PP) and (**A**,**B**) their content or (**C**,**D**) the RAPP content (Molecular mass > 2 × 10^6^ g mol^−1^) in wheat grains. SD: Standard deviation. The GluA1 nul allele is noted 0. For example, cultivars having the following HMW-GS encoded at *GluA1*, *GluB1* and *GluD1* nul, 7–8, 2–12, respectively are noted 0/78/212.

As seen in Table 2, the control of the accumulation of polymeric proteins and their molecular distribution by genetic factors is limited, in contrast to the control exerted by various environmental factors (σ^2^_G_/σ^2^_R_ = 11.10 vs. σ^2^_E_/σ^2^_R_ = 187.46 and σ^2^_G_/σ^2^_R_ = 8.90 vs. σ^2^_E_/σ^2^_R_ = 182.00 for PP and RAPP, respectively). Among the genetic factors, the allelic diversity of the glutenin subunits plays a particularly important role [21], and LMW-GS diversity contributed two times more to polymer characteristics than HMW-GS [25]. Generally, among the 14 alleles encoding HMW-GS, the *Glu-A1* null allele and the subunit *Glu-D1* 2–12 had a negative influence on polymer contents (Figure 5B,D), which is even more important as the molecular characteristics of these polymers are important (PP vs. RAPP). In contrast, the subunits *Glu-A1* 1 and *Glu-D1* 5–10 increased the polymer contents. For the 17 alleles encoding LMW-GS, *Glu-A3* a and e had a negative influence, whereas *Glu-A3* d was positively associated. For the *Glu-B3* locus, allele c was negatively associated, whereas b was positively associated with the polymer contents. The allele *Glu-D3* b was positively associated to the polymer contents (Figure 5A,C).

Our results, which underline that the glutenin association level is strongly correlated with the nature of the LMW-GS and HMW-GS present (especially HMW-GS pair 5 + 10 vs. HMW-GS pair 2 + 12 coded by *Glu-D1*), are in agreement with previous studies. As demonstrated by the different experimental approaches carried out already at the end of the 20th century [2,36,37,38], the different glutenin subunits (i.e., HMW-GS, LMW-GS, and HMW-GS *x* and *y* type) are unequally distributed within polymers. These results demonstrate the existence of a highly ordered structure in which some subunits play a predominant role, notably because of their difference in functionality (i.e., number and especially position of cysteine residues capable of forming intermolecular bonds) [3].

### 3.4. RAPP Accumulation Can Be Modulated by Glutenins Redox Status Change in Response to Growing Conditions

The accumulation of PP starts early in the grain (from 7 Days After Anthesis (DAA)) and continues up to the beginning of the desiccation phase of the grain (i.e., when grain humidity reaches ≈ 40%) [7]. According to the various physiological observations carried out since the early 2000s [39,40,41,42], a phase of an important increase in polymerized proteins, as revealed by MWD measuring, coincides perfectly with the grain desiccation phase [7], whatever the culture conditions applied (i.e., temperature and nutrient availability). The strengthening of the aggregation character in these polymeric proteins during grain desiccation results from the reinforcement of intermolecular interactions between the different glutenin subunits (HMW-GS and LMW-GS) [9,43]. During grain filling, glutenin subunits and particularly LMW-GS, which have a large amount of free SH, become oxidized during grain desiccation, which coincides with the MWD increase and the HMW polymer (RAPP) accumulation [9].

The amplitude of the general mechanism of the formation of HMW polymers (RAPP) in the grain, which therefore corresponds to a natural modification of the redox status of the grain reserve proteins during the desiccation phase, will be dependent on the potential for molecular interaction in the glutenin subunits (i.e., the number of free SH residues engaging in the formation of intermolecular SS bonds). For a given wheat genotype, any modification of this molecular interaction potential (i.e., limitation of reactive glutenin-free SH residues) can affect the formation of RAPP and, consequently, the MWD of total polymers. Among the natural molecules likely to behave as potential “interaction limiters”, we can mention glutathione, which ensures the maintenance of the redox status at a cellular level but also the storage and transport of the reduced sulfur necessary for the synthesis of proteins [44,45,46]. In fact, this tripeptide may occur endogenously in wheat grain in the form of protein-glutathione mixed disulfides (PSSG) [40,41,47,48,49,50,51,52,53,54,55,56,57,58,59,60,61]. Approximately 85% of PSSG in mature wheat grains are represented by polymeric proteins (PP) conjugated to glutathione (PPSSG) [10], and the formation of PSSG coincides with grain desiccation (HMW polymer formation) [9,10,40].

**Figure 6:**
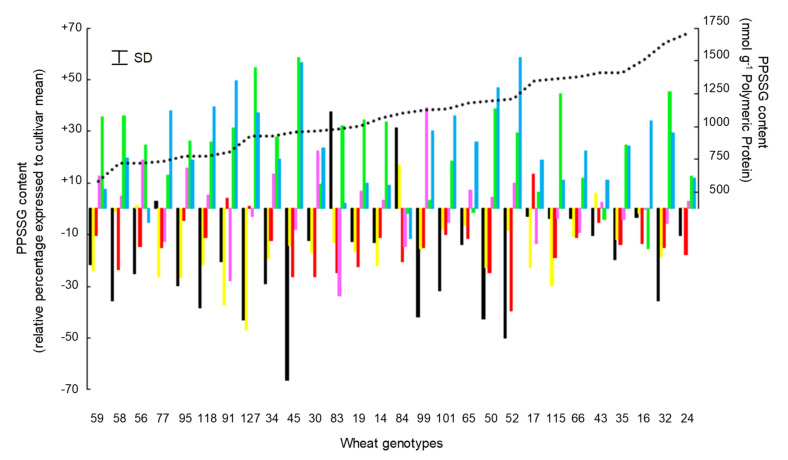
Variation in grain PPSSG content in response to modification of growing conditions represented by colored bars (■: Y1L1, ■: Y1L2, ■: Y2L4, ■: Y1L3, ■: Y2L6, ■: Y2L5—see Table S1) for a significant set of 28 wheat cultivars. Genotypic mean values (*n* = 6) of PPSSG content are represented by the dashed line. SD: Standard deviation.

In the context of our study, the polymeric protein-bound glutathione (PPSSG) content is characterized by a large amplitude of variation (i.e., from 320.5 to 1755.8 nmol GSH g PP, with 935.0 nmol GSH g PP as a mean value). As the results presented in Table 2 demonstrate, PPSSG accumulated at the end of the grain desiccation period are strongly under the control of growing conditions. Indeed, the ratio of variances (i.e., environmental variance/genetic variance) is here largely in favor of an environmental effect (σ^2^_E_/σ^2^_G_ > 12.43). The environmental control of the PPSSG content of the grains for a representative sample of 28 genotypes of wheat (representative of the allelic diversity of HMW-GS and LMW-GS) is illustrated in Figure 6. Thus, whatever the considered genotype, the growing conditions Y1L1, Y1L2, and Y2L4, which are not statistically different (Supplementary Table S3), are mainly unfavorable (i.e., compared to the mean value of the genotype) to the accumulation of PPSSG. The growing conditions Y2L6 and Y2L5 and, to a lesser extent Y1L3, are significantly favorable (i.e., compared to the mean value of the genotype) for the accumulation of PPSSG. In addition, this control is verified regardless of the average level of PPSSG accumulation in the grains.

Despite predominant environmental control for the accumulation of RAPP, some genetic control exists (Table 2 and Supplementary Table S4). As can be seen from Figure 6, the amplitude of variation of the genotypic response to environmental variation factors (i.e., PPSSG formation) is large.

Changes in PPSSG accumulation may result from significant variations in the redox status at the cellular level (i.e., normal high GSH/GSSH ratio) in response to environmental stimuli (i.e., environmental stress causing the appearance of free radicals) during grain filling. In our hypothesis, any change in cellular redox status (i.e., decrease in the GSH/GSSH ratio) upstream of the grain desiccation phase will limit the potential for protein interaction (i.e., RAPP accumulation) because of PPSSG formation, largely because of SH/SS exchanges with accumulated Glutathione oxidized (GSSG) [48].

**Figure 7:**
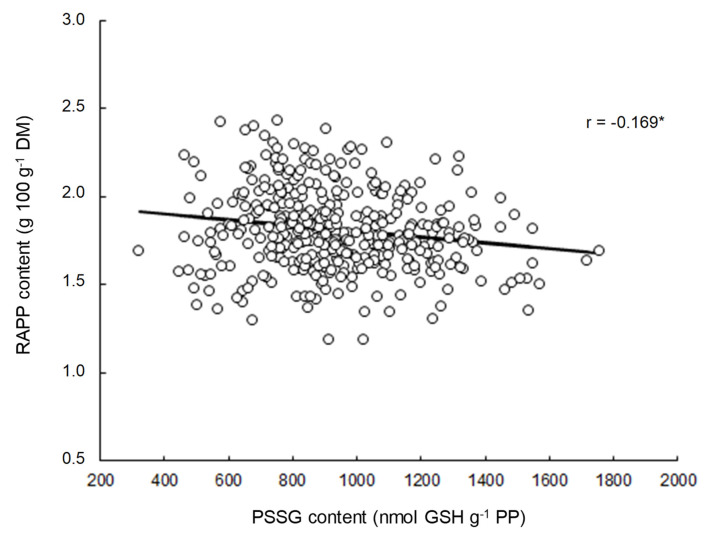
Relationship between PPSSG content and RAPP content in grains 130 wheat cultivars cultivated in six different growing conditions. Statistical significance was accepted at *p* < 0.01 (*).

Figure 7 shows the relationship between the content of PPSSG in wheat kernels and the amount of RAPP formed at maturity. As shown by the relationship calculated from 130 wheat cultivars exposed to six different growing conditions, a significant general trend (*r* = −0.169, *p* < 0.01) was observed. Thus, even if this general trend is a little weak, these first results seem to confirm our hypothesis according to which the accumulation of PPSSG may limits the formation of RAPP by playing the role of a “polymerization terminator” during the desiccation phase of the wheat grain. These initial results invite us to continue our investigations in order to completely confirm this hypothesis, with emphasis on the individual behavior of the different wheat genotypes. The general tendency can indeed mask quite divergent individual behaviors, as suggested by the results presented in Figure 6. An attempt of an illustration is presented in Supplementary Figure S2 from a limited number of genotypes. Even if the number of growing conditions studied here is too low (*n* = 6) to conclude statistical results (i.e., to obtain *r* > 0.80), the fact remains that the study of individual responses (estimated by linear equations) allows to highlight more intense relationships (i.e., *r* > −0.55 in most cases compared to *r* = −0.169 for the general trend) (Supplementary Figure S2) between the content of PPSSG and the amount of RAPP in wheat kernels.

## 4. Conclusions

The formation of gluten polymers has been and still is the subject of numerous studies because of the preponderant role they seem to play in the definition of the technological properties of flours and/or bread wheat doughs. Even if our results support this general idea, they also confirm that all the glutenin polymers do not have the same importance for the definition of the rheological properties of flours and/or doughs. In fact, based on the A4F analytical approach, our results confirm that a critical molecular mass can be defined for this protein fraction as the molecular mass above which the polymer content contributes, for example, to dough baking strength and mixing tolerance. Above a critical molecular mass of about 2 × 10^6^ g mol^−1^, the polymers (i.e., HMW polymers) can be considered as “rheologically active polymers” (RAPP).

Although genetic control cannot be ignored during the accumulation of these important HMW polymers (RAPP) in the grain, it is limited compared to the control operated by environmental factors (i.e., impact 20 times greater). Among the various hypotheses studied today to try to explain all or part of this environmental control, the variations undergone by the cellular redox status in response to environmental stimuli (i.e., various environmental stresses in response of growing conditions) upstream of the grain desiccation phase can explain the observed fluctuations in the inter-polymer interactions. Under the influence of oxidative stresses (whatever their origin), the redox status of glutathione (i.e., predominant non-protein thiol in most plant species that ensures the maintenance of this redox status) is modified and generates GSSG dimers which can interact with PP by the formation of PPSSG conjugates. The accumulation of these PPSSG conjugates reduces the formation of the RAPP by limiting the intermolecular interactions naturally set up within the PPs in the grain during the desiccation phase. By limiting the potential for the formation of RAPP, this phenomenon is therefore potentially responsible for significant changes (i.e., decrease) in the technological properties of the wheat genotypes concerned.

These first results invite us to continue our investigations to fully confirm this phenomenon, with emphasis on the behavior of wheat genotypes subjected to various growing conditions. Given the current and expected environmental impacts (i.e., climate change with increasing environmental stresses), it is essential to better understand these cellular mechanisms by exploring possible “redox” strategies [62] integrated within the patterns of genetic improvement of soft wheat cultivars to obtain durable technological properties.

## Figures and Tables

**Table 1:** Abbreviations, averages, and ranges of the five Mixogram parameters and the four Alveogram parameters used for the characterization of the 130 cultivars grown in six locations (780 samples).

Variables (Units)	Abbreviations	Mean Value	Minimum Value	Maximum Value
Mixogram parameters				
Midline Left of Peak Width (%)	MLW	17.3	3.0	56.1
Midline Peak Width (%)	MPW	15.2	2.9	37.5
Midline Right of Peak Width (%)	MRW	13.1	3.3	20.7
Midline Time × = 8 min Width (%)	MT × W	12.7	3.8	26.6
Midline Time × = 8 min Integral (% min^−1^)	MT × I	261.6	106.1	416.1
Alverogram parameters				
Dough baking strength (10^−4^ Joules)	W	214.2	43.0	563.0
Dough tenacity (mm)	P	62.3	16.0	131.0
Dough extensibility (mm)	L	104.1	36.0	240.0
Dough viscoelastic balance	P/L	0.69	0.11	2.75

**Table 2:** Genetic (G) and Environmental (E) Influence on Polymeric Protein contents determined by ANOVA (*F*-test) for 130 French common wheat genotypes cultivated in three different locations for 2 years.

Parameters	Minimum Value	Maximum Value	Mean Value	σ^2^_G_/σ^2^_R_	σ^2^_E_/σ^2^_R_	σ^2^_E_/σ^2^_G_
Total protein content ^1^	8.70	15.10	11.19	11.41 ***	271.58 ***	23.80
Polymeric protein content ^1^	2.78	5.75	4.02	11.10 ***	187.46 ***	16.88
Polymeric protein M_w_^2^	1.14	22.97	7.64	3.37 ***	38.97 ***	11.56
Polymeric protein M_w_/M_n_^3^	0.64	24.62	7.91	2.63 ***	20.62 ***	7.83
Polymeric protein R_w_^4^	1.80	70.80	31.42	5.32 ***	42.65 ***	8.01
RAPP content ^5^	0.84	3.11	1.70	8.90 ***	182.00 ***	20.45
PPSSG content ^6^	320.5	1755.8	935.0	4.40 ***	54.67 ***	12.43

^1^ Protein content in g 100 g DM. ^2^ M_w_: Weight-average molecular mass in 10^6^ g mol^−1^. ^3^ M_w_/M_n_: Polydispersity Index (PI). ^4^ R_w_: Weight-average mean square radius in nm. ^5^ RAPP content: polymeric proteins with M_w_ > 2 × 10^6^ g mol^−1^ (i.e., critical mass). ^6^ PPSSG: Polymeric Protein-bound Glutathione in GSH nmol/g polymeric protein. (***) indicates *F*-test significance at 0.1% level of probability.

## Supplementary Materials

The following are available online at https://www.mdpi.com/2304-8158/9/11/1675/s1, Table S1: Locations and Seed companies involved in multilocal trials, Table S2: Frequencies of alleles encoding HMW-GS an LMW-GS in the 130 wheat cultivars, Table S3: Impact of growing conditions on PPSSG content for the 130 wheat genotypes, Table S4: Impact of wheat genotype nature on PPSSG content for the 130 wheat genotypes, Figure S1: Relationship between polymeric protein content in wheat grains and their (A) weight-average molecular mass (M_W_), (B) weight-average mean square radius (R_W_), Figure S2: Relationship between PPSSG content and RAPP content in grains for a significant set of 28 wheat cultivars cultivated in 6 different environmental conditions.
